# Assessing the influence of conflict on the dynamics of sex work and the HIV and HCV epidemics in Ukraine: protocol for an observational, ethnographic, and mathematical modeling study

**DOI:** 10.1186/s12914-019-0201-y

**Published:** 2019-05-20

**Authors:** Marissa Becker, Olga Balakireva, Daria Pavlova, Shajy Isac, Eve Cheuk, Elizabeth Roberts, Evelyn Forget, Huiting Ma, Lisa Lazarus, Paul Sandstrom, James Blanchard, Sharmistha Mishra, Rob Lorway, Michael Pickles, on behalf of the Dynamics Study Team, Leigh McClarty, Francois Cholette, Emma Lee, Lyle McKinnon, Maureen Murney, Nicole Herpai, Sevgi Aral, Ani Shakarishvili, Stephen Moses, Tetiana Bondar, Tatiana Tarasova

**Affiliations:** 1 Centre for Global Public Health, Rady Faculty of Health Sciences, University of Manitoba, R070 Med Rehab Bldg, 771 McDermot Avenue, Winnipeg, Manitoba R3E 0T6 Canada; 2 Department of Community Health Sciences, University of Manitoba, S113 – 750 Bannatyne Avenue, Winnipeg, Manitoba R3E 0W3 Canada; 3 Ukrainian Institute for Social Research after Oleksandr Yaremenko, 26 Panasa Myrnogo Str., Of. 211, Kyiv, 01011 Ukraine; 4 India Health Action Trust, E17, Ring Road, Defence Colony, New Delhi, 110021 India; 5 University of Toronto, 27 King’s College Circle, Toronto, Canada; 6 St. Michael’s Hospital, Li Ka Shing Knowledge Institute, University of Toronto, 209 Victoria St, Toronto, ON M5B 1T8 Canada; 7 JC Wilt National HIV and Retrovirology Laboratory, 745 Logan Avenue, Winnipeg, R3E 3L5 Canada

**Keywords:** HIV, HCV, Ukraine, Sex work, Conflict

## Abstract

**Background:**

Armed conflict erupted in eastern Ukraine in 2014 and still continues. This conflict has resulted in an intensification of poverty, displacement and migration, and has weakened the local health system. Ukraine has some of the highest rates of HIV and Hepatitis C (HCV) in Europe. Whether and how the current conflict, and its consequences, will lead to changes in the HIV and HCV epidemic in Ukraine is unclear. Our study aims to characterize how the armed conflict in eastern Ukraine and its consequences influence the pattern, practice, and experience of sex work and how this affects HIV and HCV rates among female sex workers (FSWs) and their clients.

**Methods:**

We are implementing a 5-year mixed methods study in Dnipro, eastern Ukraine. Serial mapping and size estimation of FSWs and clients will be conducted followed by bio-behavioral cross-sectional surveys among FSWs and their clients. The qualitative component of the study will include in-depth interviews with FSWs and other key stakeholders and participant diaries will be implemented with FSWs. We will also conduct an archival review over the course of the project. Finally, we will use these data to develop and structure a mathematical model with which to estimate the potential influence of changes due to conflict on the trajectory of HIV and HCV epidemics among FSW and clients.

**Discussion:**

The limited data that exists on the effect of conflict on disease transmission provides mixed results. Our study will provide rigorous, timely and context-specific data on HIV and HCV transmission in the setting of conflict. This information can be used to inform the design and delivery of HIV and HCV prevention and care services.

## Background

In 2014, an armed conflict erupted, and still continues, in Ukraine. The Crimean Peninsula was annexed and the eastern regions of Donetsk and Luhansk of Ukraine were attacked [1]. This conflict has led to the internal displacement of more than 1.6 million people and has resulted in the disruption of water, sanitation, and medical services [1–3]. With a weakening public health system in the occupied and eastern regions and movement of internally displaced people into the mainland, there are growing concerns about the potential rise in HIV and Hepatitis C (HCV) in Ukraine [4–9].

By 2016, Ukraine already had the second largest HIV epidemic in Eastern Europe and Central Asia with 240,000 people living with HIV (PLHIV) and 13,000 new HIV infections for an incidence among adults 15–49 years of 0.55 per 1000 population [10, 11]. Ukraine also has some of the highest rates of HCV, with a prevalence of 12% in the general population [12]. While overall there has not been an increase in HIV incidence since the start of the conflict in 2014, some areas have seen HIV incidence increase by up to 36% from 2014 to 2016 [7, 13, 14]. In general, the HIV epidemic in Ukraine is concentrated in the east where the armed conflict is most severe [15]. Among registered PLHIV, 50% live in just three regions of eastern Ukraine (Donetsk, Dnipropetrovsk, and Odessa) with an HIV prevalence rate of 736 per 100,000 population in Dnipropetrovsk in 2014 [10, 16]. Given its close proximity of 200 km from the center of conflict in Donetsk, the city of Dnipro in Dnipropetrovsk has been one of the main destinations for internally displaced persons and members of the frontline forces since the beginning of conflict [17]. Vasylyeva et al. recently demonstrated that HIV viral lineages have moved from Donetsk and Luhansk in eastern Ukraine to other parts of Ukraine, corresponding to the movement of people displaced during the armed conflict [7].

Conflict creates “complex emergencies” [18, 19] with consequences which include an intensification of poverty, displacement and migration, and weakened health systems [4, 5, 19–21]. Despite our understanding of the linkage between poverty [22–24], violence [25, 26], and migration with HIV [27, 28], there are insufficient data to conclude that conflict leads to higher rates of HIV [20, 21]. Two systematic reviews found no evidence to suggest that there was an increase in HIV during conflict [20, 21]. However, the authors caution that the association between conflict and HIV is complex and that this association needs to be examined in context.

In effort to understand these complex relationships further, Mock et al. developed a framework on conflict and HIV which uses a social ecology approach and examines the interactions of the multiple causes of an HIV epidemic, at the individual, household and community level, and emphasizes the dynamic nature of these factors [29]. This framework is useful for better understanding the interrelationship between conflict and HIV and illustrates how many of the factors present in Ukraine right now, such as migration, increased interaction among military and civilians, increased population mixing, fragmentation of families, and increased levels of commercial and casual sex, are some of the factors which have the potential to lead to an increase in HIV [8, 29–31].

.In Ukraine, there are an estimated 80,100 female sex workers (FSWs) with an HIV prevalence of 5.2% in 2016 [11, 14, 32]. Very little data exist on whether sex work changes during times of conflict. Elmes et al. began to address this question in Zimbabwe following the economic upheaval in the country and demonstrated that the changing economic conditions there resulted in women exchanging sex in less formal settings and engaging in riskier transactions [33].

To our knowledge, no data exists on the influence of conflict on HIV in Eastern Europe, nor on the influence of conflict on sex work in the region. Our study aims to characterize how the armed conflict in eastern Ukraine and its consequences influence the pattern, practice, and experience of sex work and how this affects HIV and HCV rates among FSWs and their clients.

## Methods

### Study setting

The study will take place in Dnipro, an industrial city in eastern Ukraine, with a population of approximately 1 million [34]. Dnipro was chosen for this study due to its proximity to the center of the conflict (200 km), its HIV prevalence among FSWs (6.7%) [35], and the size of the sex work population [1087 (range 817–1357)] [36]. The study will be conducted in partnership with the Ukrainian Institute for Social Research after Oleksandr Yaremenko (UISR) and The Center for Public Health of the Ministry of Health in Ukraine.

### Study hypothesis and objectives

We hypothesize that conflict and its consequences are associated with changes in the organization of sex work (more people selling and buying sex), sex work patterns (earlier age of entry into sex work), and the practice and experience of sex work (higher turnover, more clients, more injection drug use, and more violence), leading to increased HIV and HCV transmission among FSWs and clients.

Our overarching objectives are:

i) To characterize how conflict and its consequences influence the organization (where and how sex work is managed, FSW/client population size), pattern (age distribution, sex work duration), practice and experience of sex work (client volume, drug use, violence), uptake of health and social services and the HIV and HCV prevalence among FSWs and clients in Dnipro.
ii) To estimate the influence of political conflict and its consequences via changes in sex work on HIV and HCV transmission among FSWs and clients.

### Study design

**Fig. 1:**
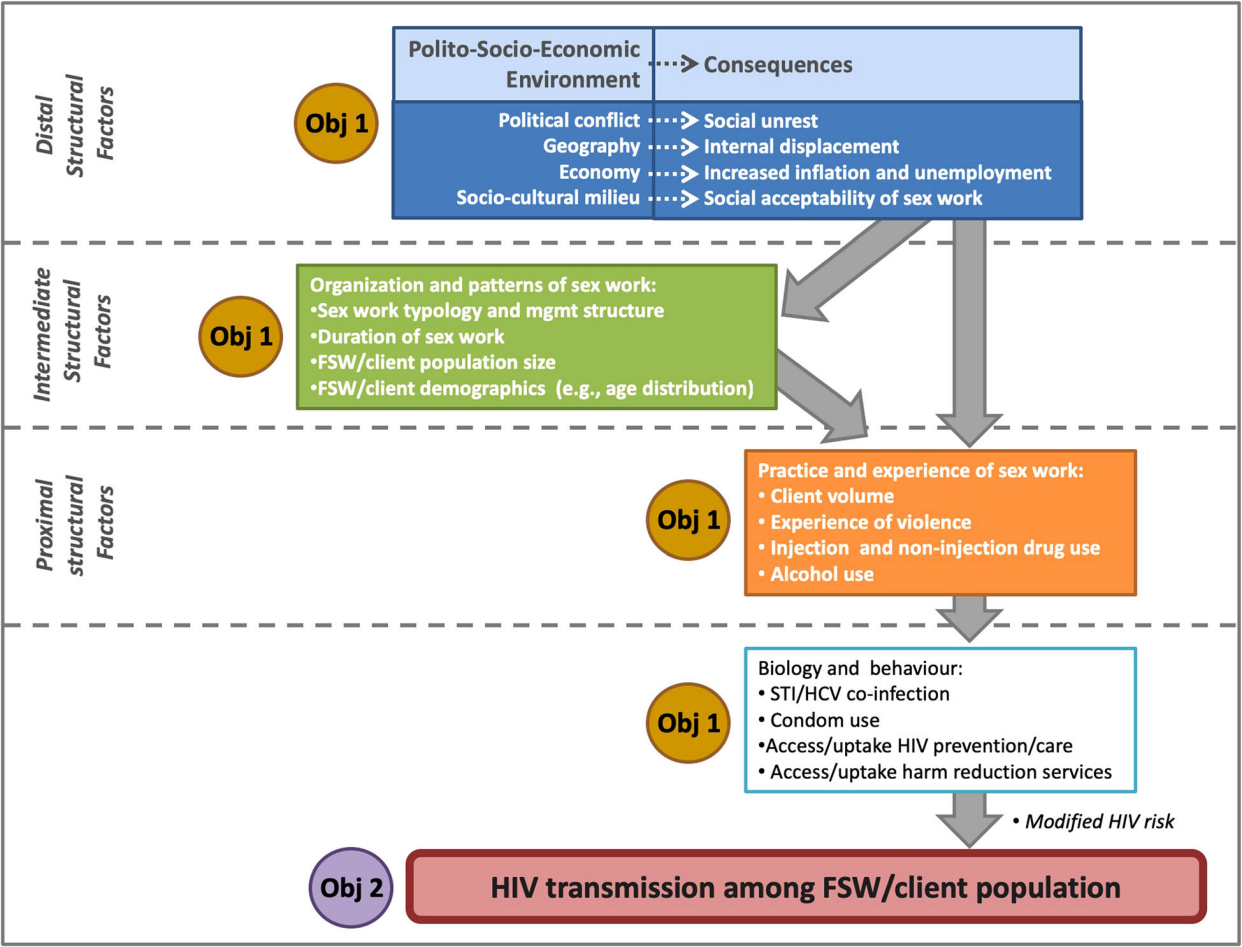
Conceptual Framework for HIV transmission

This study is a serial cross-sectional biological and behavioural survey and it does not offer or assign participants to any health-related interventions. We will use a mixed methods approach with both quantitative and qualitative methodologies to address these objectives (Fig. 1). These methods include: i) mapping; ii) bio-behavioural surveys; iii) in-depth interviews, participant diaries, and archival review; and iv) mathematical modelling.

Serial mapping and estimation of FSW and clients will be conducted 3 times at 2-year intervals to measure changes in the organization of sex work. Serial bio-behavioral cross-sectional surveys will quantify population-based changes in the pattern, practice, and experiences of sex work; and uptake of health and social services; and HIV/HCV prevalence. The qualitative component of the study, including the in-depth interviews, participant diaries, and archival review will explore the influence of conflict at the individual level and how this changes over time. Finally, we will use these data to develop and structure a mathematical model with which to estimate the potential influence of changes due to conflict on the trajectory of HIV and HCV epidemics among FSW and clients.

#### Mapping and enumeration

First, UISR will conduct geographic mapping [37] of venues associated with sex work (‘hotspots’ defined as locations where FSWs solicit and/or have sex with clients) and characterize the attributes of hotspots. Examples of these attributes include whether the spot is considered safe, whether it is a spot where injection drug use occurs, or where people who inject drugs congregate. We will simultaneously estimate the FSW and client population size within hotspots and by typology of sex work in Dnipro. Our team has extensive experience with geographic mapping and key population size estimation [37–41]. FSW and client population size estimates by hotspot will comprise the sampling frame for the cross-sectional bio-behavioral surveys among FSWs and clients [38]. Sampling of FSWs and clients will follow a two stage sampling design, where in the first stage a representative sample of hotspots will be selected, followed in the second stage by randomly sampling FSWs or clients from each selected hotspot. To generate a representative sample of hotspots we will use a stratified random sampling, where the hotspots are selected randomly after stratifying the hotspots by administrative division and type of hotspot. The sample size for each selected hotspot is proportional to the size of the respective population estimated in the hotspot from the mapping. Recruitment of clients will be based on time-location sampling.

#### Serial cross-sectional bio-Behavioural surveys

**Fig. 2:**
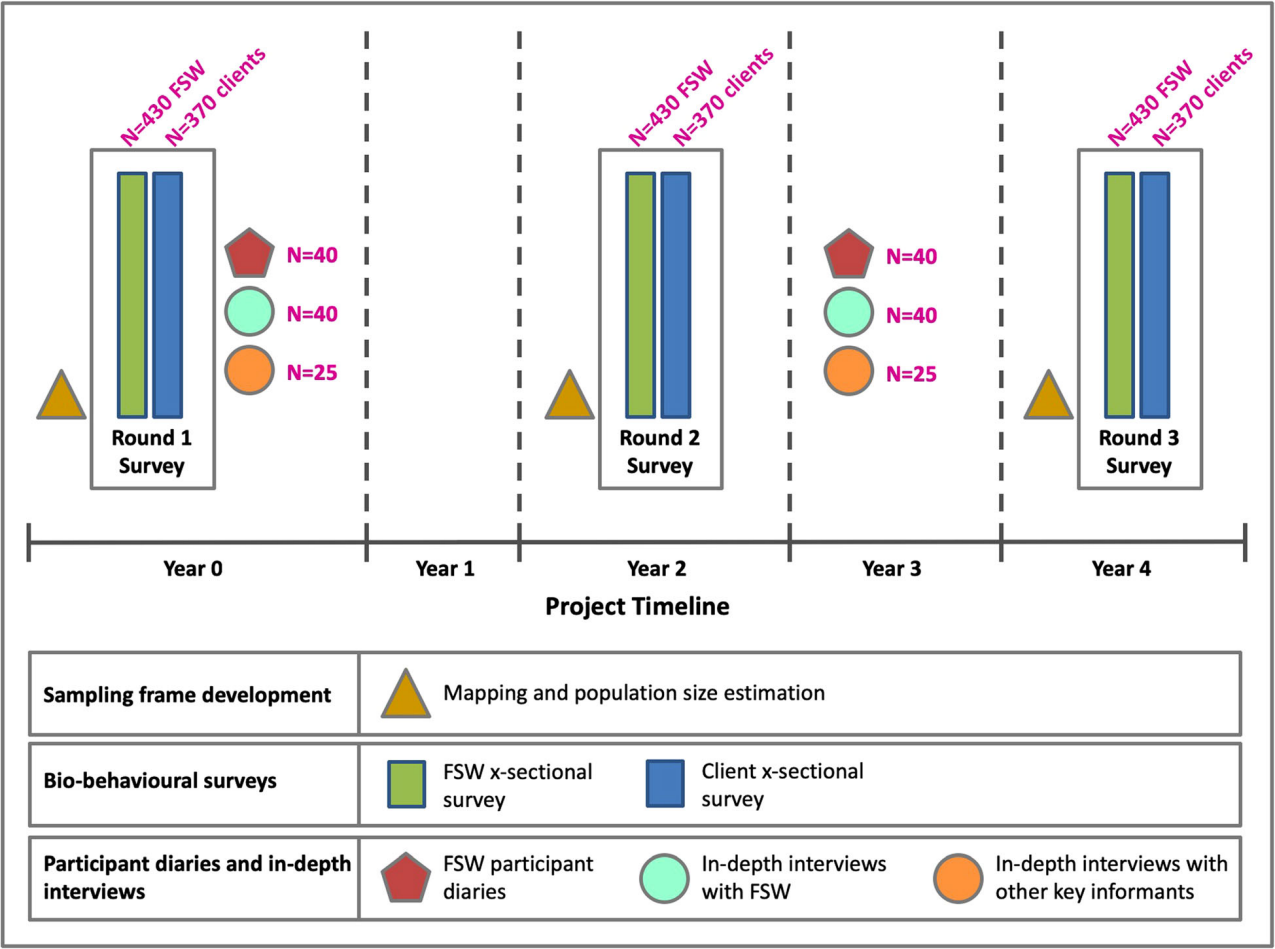
Study design and methodology

We will conduct three rounds of a cross-sectional bio-behavioral survey among FSWs and clients at three time points approximately 2 years apart (Fig. 2). Each round of survey is anticipated to take 3 months.

FSWs age 14 years or older who self-identify as currently engaged in sex work are eligible to participate. Eligible clients will be men aged 18 or over who have ever purchased sex.

Recruitment of participants (FSWs and clients) will be facilitated by outreach workers who will act as mobilizers at the selected hotspots. These mobilizers will visit the selected hotspots and explain the study. If a potential participant expresses interest in the study, s/he will be escorted to a mobile van in the vicinity where s/he will meet with a trained interviewer. These interviewers will obtain written informed consent followed by face-to-face administration of a structured questionnaire in the local language (Russian). Survey questions, based on the Integrated Biological and Behavioural Survey (IBBS) Guidelines and our past work [42], have been piloted and locally validated. Participants will be asked about basic socio-demographic characteristics including age, educational level, and marital status; vulnerabilities and protective factors related to HIV and HCV risk, including partner volume, condom use, and injection drug use; and structural factors including experience of violence, alcohol consumption, and access to programs and clinical services. Questions specific to the conflict for FSWs include: interactions with military, migration resulting from conflict, economic impact from conflict, and entry into sex work as a result of the conflict.

The FSW questionnaire is anticipated to take 45 min to complete. The client questionnaire is anticipated to take 25 min to complete.

Following administration of the questionnaire, consenting participants will receive HIV and HCV rapid testing conducted by a medical worker certified by the Dnipropetrovsk Oblast AIDS Centre. Pre- and post-test counselling will be provided according to national guidelines [43, 44]. Participants can choose not to receive testing results. All participants will be linked with local HIV and HCV prevention programs and participants who test positive will be referred to treatment services. A dried blood spot sample (DBS) will also be collected and sent to the National HIV and Retrovirology Laboratories, Winnipeg, Canada for serological confirmation and HIV/HCV viral sequencing analysis, if consent is given. Participants will be able to access their results of the reference DBS HIV and HCV serological test results through a toll-free hotline number established for this study. This hotline was created as a result of request voiced by the community and will be managed by the Dnipropetrovsk Oblast AIDS Centre.

### Sample size for the serial cross-sectional bio-behavioral surveys

The target sample size for each round of surveys is 560 FSWs and 370 clients based on 80% power and a 5% alpha error in two outcomes: experience of physical violence among FSW during encounters with clients (current level 42%) [32, 35] and injecting drug use among clients (current level 26%) [45]. This will also account for a 10% ineligibility, observed in our previous work in Dnipro. The sample size accounts for a design effect of 1.5 via the cluster-based sampling.

#### In-depth interviews, diaries, and archival review

Qualitative data will be collected at two time points approximately 3 years apart. Three different qualitative methodologies will be used: i) in-depth interviews, ii) participant diaries, and iii) archival review.

### In-depth interviews and participant diaries

Forty FSWs will be recruited through two main routes: i) from survey participants who have expressed interest in taking part in the qualitative component; and ii) purposively recruited through networks of “social workers” (a type of local community health worker) with whom FSWs have established rapport and trust. In each wave of data collection, participants will be interviewed every 2 weeks over a period of 2 months. Interviewers will be affiliated with UISR and will receive training on the semi-structured interview guide and qualitative interviewing techniques. Participants will be invited to also take part in diary writing – 4 entries per week throughout the course of their interview participation. The first interview of this qualitative series will elicit participants’ perceptions in the following areas: of the sex trade generally; their experience with clients, coworkers, and police; migration; their health care needs and access; and how these aspects of their lives have been influenced by conflict. Two weeks later during the second interview, discussion will center around topics to be covered in the diaries: 1) work life, income, and trying to make ends meet; 2) specific interactions with clients and other people in the industry in the past 2 weeks that carried important meaning to the participants; 3) any experiences of accessing health and social services; and 4) interactions in their personal and/or family lives, including those with partners and children. During the second interview, participants will be asked if they would reflect and expand on topics discussed in the first interview. Discussion during the third and fourth (exit) interviews will revolve around, and delve more deeply into, the impressions and events recorded by the participants in their diaries. Each interview is expected to last approximately 1 hour and will be audio recorded. Following the transcription of diary entries, participants will have the opportunity to have their diaries returned to them.

Additional in-depth interviews will be conducted with key informants: brothel managers or pimps, service providers (e.g., medical workers/clinicians and social/outreach workers who provide services to FSWs), law enforcement officials (e.g., both national and regional levels that deal with issues related to sex work, human trafficking, and human rights) and other national stakeholders (e.g., representatives from Ministry of Health and Ministry of Internal Affairs, NGOs that work with issues related to HIV and human rights). Participants for key informant interviews will be purposively sampled. Social workers will assist in recruiting key informants associated with sex work such as brothel managers and pimps. Service providers and national stakeholders will be recruited through existing relationships held by the study team. These interviews will aim at gathering information on the social, political, and policy environments surrounding sex work. Twenty-five interviews will be conducted. Interviews are expected to last approximately 1 hour and will be audio recorded.

### Archival review

Data from the qualitative strand will be triangulated with primary archival data, such as newspapers, parliamentary debate transcripts, and municipal records from the State Statistics Service of Ukraine, the Ministry of Health of Ukraine, and country-level behavioural surveys. This data will establish the social, geopolitical, and economic milieu in which the experience of individual FSW/client is embedded as conflict continues to evolve.

### Analysis

#### Quantitative data analysis

Changes in the organization of sex work (ecological-level outcomes): We will use the serial mapping data to identify variability – at the ecological level - in the organization of sex work (defined as distribution of sex work typologies) and absolute and relative population size of FSWs and of clients between the survey time-points.

Changes in the pattern and practice of sex work: We will examine changes in the pattern and practice of sex work by using both ecological and individual level variables. This data will be obtained from the bio-behavioural survey as well as publicly available data. The exposure variables are as follows:

- *Ecological* exposure variables include established measures of conflict-related factors (e.g, numbers of troops in DNK and battlefield deaths), internal displacement, inflation and unemployment rate, in the year of each survey [20, 21, 46]. *Individual-level* exposure variables include known consequences of political conflict, such as household income, recent unemployment, and migration status, which will be collected from the study survey [46, 47].

Outcome variables for the analysis include the following:

- *Organization* of sex work (ecological outcomes): sex work typology (place/method of solicitation); FSW/client population size. The *pattern* of sex work will be examined by looking at the following individual level outcomes: age of entry into sex work, duration in sex work. The *practice* of sex work will be examined with the following individual level outcomes: FSW-reported client volume; drug use; FSW experience of different types of violence (in the preceding month).

We will examine socio-demographics, individual-level exposures, and outcomes using measures of central tendency, and compare outcomes between rounds using parametric and non-parametric tests of difference, with *p* < 0.05 for significance.

Multivariate logistic regression will be used for dichotomous outcomes, linear regression for continuous outcomes, and Poisson regression for count outcomes. Multi-level models will be used to include both ecological and individual outcomes. Each outcome will be analyzed separately for FSWs and clients. Only ecological outcomes will be used to examine sex work organization. All outcomes will be tested as potential covariates for the HIV/HCV prevalence outcomes. Adjustments will be made for age and a proximate determinant framework of nested models will be used to isolate the individual-level mediators of HIV/HCV prevalence.

#### Qualitative data analysis

All qualitative data will undergo interpretivist analysis after being transcribed and translated into English. The data will be analyzed using an idiographic approach. Interview recordings and diaries will be examined individually and notes will be made. Inductive thematic analysis will use these notes to construct conceptual themes from the qualitative data and the archival texts. The data will then be imported into NVivo12 Plus software to complete content analysis. This software will examine the frequency of themes, the characteristics of each theme, and will sort themes into meta-themes/sub-themes. Aggregated data patterns will be used to demonstrate change over time. Data from the interviews and diaries will be triangulated with the archival data.

### Mathematical modeling

A mathematical model of HIV and HCV transmission among FSWs and their clients will be developed. The model structure will capture the influences identified through the cross-sectional surveys, in-depth interviews, and participant diaries and thus will reflect the empirical understanding of how conflict may be changing sex work and associated HIV and HCV risk in Dnipro. The model will incorporate the effects of structural factors both through modifying parameters linked to state-transitions, and through stratifying by exposure to the structural factor itself when the dynamics of exposure is important. These structural factors will influence risk and protective behaviours that in turn modify the individual-level probability of HIV/HCV susceptibility and/or infectivity. The model will be parameterized and calibrated using data both from the current study and the earlier Transitions study also carried out in Dnipro [36], as well as prior cross-sectional bio-behavioural surveys among FSWs [35].

The model will quantify the impact of changes in the organization, pattern and practice of sex work on the HIV/HCV epidemics among FSWs and clients from 2014 (start of the conflict) to 2022. To do this, we will use counterfactual scenarios where all parameters are as in the full model, except for key associations in the proposed causal pathway which are ‘turned off’. Impact is then calculated as the difference in cumulative infections between the full and counterfactual model projections. We will then project how the long-term, future epidemic trajectory has changed due to the conflict, using estimates from the full model over the subsequent 10 years. Finally, the model will be used to understand which conflict-mediated changes may have had the greatest epidemic impact, with a view to informing and improving HIV/HCV prevention and treatment programs.

### Data storage, security, and confidentiality

The consent forms will be stored separately from other data in a locked and secure cabinet in Kiev in the UISR office. Study forms and surveys will be transferred to Kiev where they will be kept in a secure cabinet in the UISR office. Only the Principal Investigators and Co-Investigators have access to the completed forms. Each participant is given a unique alphanumeric code for identification. All data and biological samples are associated with this Study ID only. Information is entered into a database in the UISR office in Kiev and is then uploaded to a secure server at the Centre for Global Public Health (CGPH), University of Manitoba, Canada. Interview recordings and translated interview transcripts and diaries will also be uploaded to the server from the UISR office. The CGPH network is protected by an electronic firewall and a virtual private network (VPN) double password login system. Access to the data on the secure server is granted only to study investigators and the University of Manitoba Research Ethics Board (REB). Internet access, personal e-mail access, CD writers, and USB drives are prohibited in the data laboratory. Remote access to the data server may be made available to those approved on the study team. However, all data analysis will be performed in the virtual interface and no data will be allowed to be exported from the virtual environment.

### Ethical approval

Ethical approval was obtained from the Human Research Ethics Board at the University of Manitoba [HS20653(H2017:097)], Canada, the Ethical Review Committee of the Sociological Association of Ukraine, and the Committee on Medical Ethics of the L. Gromashevsky Institute of Epidemiology and Infectious Diseases at the National Academy of Medical Sciences of Ukraine.

### Knowledge translation

We have partnered with two local community-based organizations in Dnipro that provide services to people who use drugs and sex workers so that study outcomes may lead to tangible action in policy and programs at the local level, Virtus and Road to Life. In addition, we have also partnered with the Dnipro Oblast AIDS Centre for support on HIV and HCV testing and care. At the national level, key partners include UISR, Alliance for Public Health and Center for Public Health of the Ministry of Health in Ukraine. This study will follow an integrated knowledge translation model [48] to ensure an iterative process for knowledge uptake and the refinement of program-driven research questions. During the course of the study, regular video/web conferences, in-country joint dissemination, and consultative program design meetings will be held to share study findings, refine study tools, and foster discussion around the significance and context of the findings. We will create briefing reports for national and international decision-makers. Incoming data will be integrated throughout the study to inform changes in the questionnaires and interview guides.

## Discussion

There is a strong belief that conflict increases the spread of HIV and HCV [49]. Conflict affects public health systems, impeding treatment and prevention activities and posing a major challenge to accurate epidemic monitoring; it can also lead to increased displacement and migration and is likely to affect the nature and practice of sex work, all of which will modify sexual networks. However, to date what little data there is to quantify the effect of conflict on disease transmission presents a mixed picture. Two studies in sub-Saharan Africa found no significant overall effect of conflict [20, 21], although the lack of observed effect may be due to the difficulty in collecting representative data samples at multiple time-points during conflict, including amongst groups most affected by the conflict, and hence any such result requires careful interpretation [20]. We need a better understanding of whether, and how, conflict affects the HIV epidemic, to be able to more effectively adapt and supplement prevention and treatment efforts during conflicts.

In this study we will investigate how conflict and its consequences impact the HIV and HCV epidemics in Dnipro, a city close to the conflict region in Ukraine. We will use a mixed methods approach to gain a broad understanding of how conflict influences the organization, pattern, and practice of sex work, as well as how the interplay of these factors determines the trajectory of the. The quantitative findings will be complemented and enriched by the qualitative work that will construct a narrative of health and conflict from social, geopolitical, and economic perspectives, serving as the backdrop for the unfolding HIV/HCV epidemics and public health response. The proposed qualitative work will also generate rich depictions of the lived experiences of sex workers and the effect that conflict has on their lives. Mathematical modelling will allow us to project possible trajectories of the HIV and HCV epidemics as the conflict evolves, and provide quantitative estimates of how conflict has impacted the HIV and HCV epidemics.

### Limitations

This study has several limitations. Firstly, all behavioral data is self-reported and collected face to face, and thus social desirability bias may lead to under-reporting of certain risk behaviors. Survey questionnaires have been designed to reduce the magnitude of this bias as will the inclusion of skilled interviewers. Secondly, there could be high attrition of FSWs participating in the diaries. To decrease the effect of attrition, regular contact will be maintained between participants and the research team. Finally, as the bio-behavioural surveys are cross-sectional, we will be unable to confer causality. However, we specifically chose these methods as we believe that a cross-sectional approach will give us an understanding of the broader sex worker population. We will apply a mixed-methods approach using findings from the survey coupled with more nuanced and deeper understandings at the individual level from the interviews and diaries. Taken together, and with the findings from the modelling, these methods will allow us to develop an understanding of epidemic pathways resulting from the conflict.

In the current study we have a unique and timely opportunity to understand whether and how conflict and its consequences influence HIV and HCV among FSWs, clients, and the broader population in Ukraine as the conflict unfolds. The mixed method approach used in this study will give complementary insights into the effects of conflict that will provide a stronger evidence basis for attribution than time trends in HIV prevalence alone, and the study will importantly examine explicitly how the nature and practice of sex work is modified by the conflict. Rigorous and context-specific data on HIV and HCV transmission in the setting of conflict is critical to inform prevention and care services, both currently in the Ukraine and for future conflict response in other settings.

## Abbreviations

CGPH: Centre for Global Public Health
DBS: Dried blood spot
FSWs: Female sex workers
HCV: Hepatitis C virus
IBBS: Integrated Biological and Behavioural Survey
PLHIV: People living with HIV
REB: Research ethics board
UISR: Ukrainian Institute for Social Research after Oleksandr Yaremenko
VPN: Virtual private network
